# Injury death certificates without specification of the circumstances leading to the fatal injury – the Norwegian Cause of Death Registry 2005–2014

**DOI:** 10.1186/s12963-018-0176-2

**Published:** 2018-12-24

**Authors:** Christian Lycke Ellingsen, Marta Ebbing, G. Cecilie Alfsen, Stein Emil Vollset

**Affiliations:** 1 Norwegian Institute of Public Health, PO Box 973, Sentrum N-5808, Bergen, Norway; 2 Department of Pathology, Akershus University Hospital, PO Box 1000, N-1478, Lørenskog, Norway; 3 Faculty of Medicine, University of Oslo, PO Box 1078, Blindern N-0316, Oslo, Norway; 4 Department of Global Public Health and Primary Care, University of Bergen, PO Box 7804, N-5018, Bergen, Norway; 5 Stavanger University Hospital, PO Box 8100, N-4068 Stavanger, Norway; 6 Haukeland University Hospital, PO Box 1400, N-5021 Bergen, Norway; 7 Institute for Health Metrics and Evaluation, 2301 Fifth Ave., Suite 600, Seattle, WA 98121 USA

**Keywords:** Cause of death, Accidental falls, Hip fractures, Garbage code, Redistribution, X59

## Abstract

**Background:**

For injury deaths, the underlying cause of death is defined as the circumstances leading to the injury. When this information is missing, the ICD-10 code X59 (Exposure to unspecified factor) is used. Lack of knowledge of factors causing injuries reduces the value of the cause of death statistics. The aim of this study was to identify predictors of X59-coded deaths in Norway, and to assess methods to identify the true underlying cause of injury deaths.

**Methods:**

We used data from the Norwegian Cause of Death Registry from 2005 to 2014. We used logistic regression to identify determinants of X59-coded deaths. For redistribution of the X59 deaths, we used a multinomial logistic regression model based on the cases where injury circumstances were known. The data were divided into training and test sets. The model was developed on the training set and assessed on the test set before it was applied to the X59 deaths. The models used death certificate information on the nature of injury and demographic characteristics as predictor variables. Furthermore, we mailed a query to the certifying physicians of X59 deaths reported in the year 2015, where we asked for additional information on the circumstances leading to the fatal injury.

**Results:**

There were 24,963 injury deaths reported to the Cause of Death Registry of Norway 2005–2014. Of these, 6440 (25.8%) lacked information on the circumstances leading to the death. The strongest predictor for a X59 death was the nature of injury (hip fracture), followed by lack of information on the scene of injury. Applying our redistribution algorithm, we estimated that 97% of the X59-coded deaths were accidental falls. The strongest covariate was the nature of injury, followed by place of death and age at death. In 2015, there were 591 X59-coded deaths. Queries were sent to the certifying doctors in 559 cases. Among the informative replies to the query, 88% of the deaths were reclassified to accidental falls.

**Conclusions:**

A large proportion of injury deaths in Norway lack information on the circumstances leading to the fatal injury. Typically, these deaths represent accidental falls causing hip fracture in elderly individuals.

## Background

According to the Global Burden of Disease Project, about 4.7 million (8%) deaths worldwide are caused by injuries [1]. In Norway, this represents about 2500 deaths (6% of all deaths) [1]. One of the main purposes of the classification of causes of death is to give information relevant to prevention programs and planning of health care services [2]. According to the instructions from the World Health Organization (WHO), when the cause of death is an injury or other effect of an external cause, the circumstances that gave rise to that condition should be selected as the underlying cause of death [3]. The reason for this is clear: the same anatomical injury (e.g., a skull fracture) can arise in numerous situations (for example traffic accidents, falls, and interpersonal violence), each with their own risk factors and targets for prevention. When there is insufficient information on the death certificate about the circumstances for an injury, “Exposure to unspecified factor” (ICD-10 code X59) is used as the underlying cause of death. In an ICD-10 update in effect since 2006, X59 was subdivided into X59.0 (“Exposure to unspecified factor causing fracture”) and X59.9 (“Exposure to unspecified factor causing other and unspecified injury”).

In general, when the information on the death certificate is insufficient to identify the true underlying cause of death, the death will be classified using uninformative codes. The term “garbage codes” was introduced by Murray and Lopez in 1996 to describe such codes [4]. In order to get a better epidemiological overview and be able to compare cause of death statistics in different countries and over different periods, there have been attempts to identify which informative causes of death (target groups) the garbage code deaths statistically represent [5]. The most comprehensive work has been carried out within the framework of the Global Burden of Disease Project [1, 4–7].

X59 is a typical example of a garbage code. The use of this code in cause of death statistics varies greatly among countries. In a study by Lu et al. from 2007, the proportion of unintentional injury deaths coded with X59 varied from 7 to 33% in the four countries included in the study [8]. The cause of death statistics have low quality if a large proportion of deaths are assigned X59. Bhalla et al. argued that the data concerning injury deaths were good if less than 20% of the deaths were assigned a garbage code, and found that in this respect only 20 out of 83 countries had high-quality data [9].

Several studies have directly or indirectly shown that a significant proportion of unspecified injury deaths represent accidental falls in the elderly [10–13].

### Aim

The aim of this study was to explore the use of the ICD-10 code X59 for injury deaths lacking information on external cause in Norway during 2005–2014. First, we wanted to find characteristics for the use of X59 as underlying cause of death in Norway. Second, using deaths with known external cause of death, we aimed to develop a classification algorithm to place the X59 deaths in the most appropriate external cause groups (target groups), and finally, compare the results of the redistribution with a query to the certifying doctors in Norway regarding the X59-coded deaths for the calendar year 2015.

## Methods

The Norwegian Cause of Death Registry contains individual data on all deaths among Norwegian residents in Norway and abroad, and, starting in the year 2012, information on deaths among foreigners who died in Norway [14]. The registry uses the IRIS software [15] with the Automated Classification of Medical Entities (ACME) module [16] for semiautomatic coding. ACME applies the rules in ICD-10 for selection of the underlying cause of death [3]. We used data from the Norwegian Cause of Death Registry for all deaths among Norwegian residents with an external cause of death for the years 2005–2014 (*N* = 24,963). From the information available, we used the following variables: calendar year of death in two categories (2005–2009 and 2010–2014), age in 10-year groups, sex, underlying cause of death (ICD-10 code), the nature of injury (ICD-10 code), the place of death, the scene of injury, and whether an autopsy (forensic or medical) was performed. The categories for underlying cause of death, the nature of injury, and the place of death are shown in Table 1. Where there was more than one injury registered on the death certificate, we used the injury considered as most serious according to the priority list in ICD-10 (main injury) [3]. We chose not to include deaths coded with Y34 (“Unspecified event, undetermined intent”) in the X59 group, as Y34 was used only two times during the entire study period, and codes in the range Y10–34 were used only 15 times. Information on the place of occurrence of the injury was missing in 40% of the deaths, so we decided to use this as a dichotomous variable to indicate whether that information was available or not.

**Table 1:** Categories of external underlying cause of death, nature of injury, and place of death

External underlying cause of death	ICD-10 codes
1. Road traffic accidents	V00 – V89.9, Y85.0
2. Accidental falls	W00 – W19.9
3. Accidental poisonings	X40 – X49.9
4. Other accidents and events of undetermined intent	V90 – V99, W20 – X39.9, X50 – X58, Y10 – Y84, Y85.9 – Y86, Y87.2 – Y89.9
5. Exposure to unspecified factor (X59)	X59, X59.0, X59.9
6. Intentional self-harm (suicide)	X60 – X84, Y87.0
7. Assault (homicide)	X85 – Y09, Y87.1
Nature of injury	
1. Head and neck injuries	S00 – S19.9
2. Thoracic injuries	S20 – S29.9
3. Injuries to abdomen and pelvis	S30 – S39.9
4. Injuries to hip and thigh	S70 – S79.9
5. Other mechanical injuries, multitrauma	S40 – S69.9, S80 – T14.9
6. Poisoning	T36 – T65.9
7. Suffocation/drowning	T17 – T17.9, T71, T75.1
8. Other injuries, sequelae	T15 – T16, T18 – T35.7, T66 – T70.9, T73 – T75.0, T75.2 – T98.3
Place of death	
1. At home	At home
2. Hospital	Somatic and psychiatric hospitals
3. Nursing home	Nursing homes, other health care institutions
4. Other known	Other known, during transport
5. Unknown	Unknown, abroad

We retrieved tabular cause-of-death data at the ICD-10 three- or four-character level for the years 2005 to 2015 for all available countries from the WHO Mortality Database [17]. For each location, we calculated the mean fractions of all external causes of death (ICD-10 code V01-Y98.9) coded with X59 (X59, X59.0 or X59.9) and with Y34 (Y34 or Y34.0) over the available years.

### Predictors for X59 as the underlying cause of death

We used multiple logistic regression to study predictors of X59 coded deaths. The explanatory variables were age, sex, nature of injury (eight categories), place of death (five categories), knowledge about the scene of injury (yes/no), whether an autopsy was performed (no autopsy, forensic autopsy, and medical autopsy) and calendar year of death in two groups. We used six age groups – below 50 years, 10-year groups up to 89, and 90 and above – in order to have sufficiently large groups.

First, we investigated each independent variable alone (univariate) before we entered all variables into a multiple predictors model. All the variables except calendar year of death had a significant effect in the univariate analyses. We used a stepwise approach in developing the final model, keeping the variables that had a significant explanatory value based on likelihood ratio, and using a *p* value of less than 0.10 as a guideline. The effects are shown as odds ratios with 95% confidence intervals. For each variable, the likelihood ratio statistic (− 2 log likelihood) and two-sided *p* values are shown.

### Redistribution of X59 cases to specific external cause groups

Redistribution is the process of reclassifying the cases with garbage codes to more informative causes of death (target groups). We developed a multinomial logistic regression model [18] with the same set of covariates as in the prediction model, except for age, where we used all 10-year age groups in the categorical variable. In contrast to the X59 deaths, a substantial number (44%) of the other injury deaths occurred in persons below 50 years. The age profile varied between the different cause groups as well, so we retained all the age groups below 50. As target groups, we used the following categories: road traffic accidents, accidental falls, accidental poisonings, other accidents and events of undetermined intent, intentional self-harm (suicide), and assault (homicide). The choice of target groups was based on the observation that accidental falls, accidental poisonings, road traffic accidents, and suicides are the largest groups of external causes of death in Norway. We chose to include intentional injuries (suicides and homicides) as well as unintentional injuries among the target groups to allow for the possibility that some of the X59 deaths could be redistributed to intentional injuries. In addition to the groups mentioned above, there are a number of small groups of injuries which were gathered in “Other/unspecified”. We used road traffic accidents as the reference outcome.

To develop a redistribution algorithm, we first excluded the deaths with X59 coded as the underlying cause of death from the dataset. The remaining 18,523 cases were randomly split into a training dataset (67%) and test dataset (33%). We then developed a multinomial regression model on the training dataset and applied it to the test dataset. For each death, we chose as the target code the external cause group with the highest probability, regardless of level.

The performance of the model on the test dataset was evaluated by calculating overall accuracy and Cohen’s kappa. If the distribution is unbalanced, with the majority of cases in one group, unadjusted overall accuracy will be artificially high, and kappa will give a more conservative measure [18]. We also calculated likelihood ratio for the difference between the full model and reduced models where we excluded one variable at a time. For each target group, we calculated sensitivity and specificity for a case being placed in this group versus all other groups. Then we applied the model on the X59-coded deaths to predict the target group for each individual death. This process was repeated 1000 times with new separation into training and test datasets, and the median and inter-quartile range of the results were calculated.

### Query to the certifying doctors

**Fig. 1:**
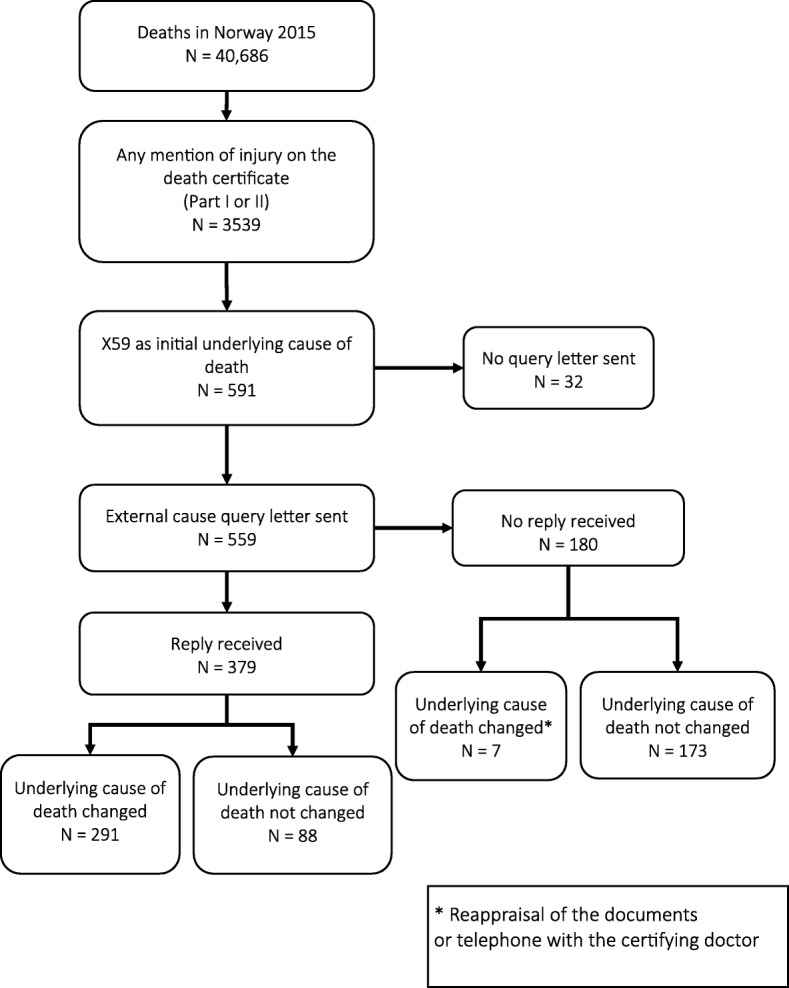
Query to the certifying doctors regarding X59 cases in Norway, 2015

As part of the regular operation of the Norwegian Cause of Death Registry, a quality assurance project was carried out during 2015–2016 on deaths coded X59 that occurred in the calendar year 2015. A query letter was sent to the certifying doctors for X59-coded deaths, collecting information about the circumstances of the injury. We used the replies to identify the external factor causing the death. By the end of the year 2016, we identified 32 additional cases where no query letter had been sent. In addition, we searched for cases where another type of query letter had been sent, and where the underlying cause of death was X59 (see Fig. 1).

### Statistical analysis

For statistical analysis, we used the R software [19]. For binary logistic regression, we calculated odds ratios with 95% confidence intervals, likelihood ratio statistics (−2LL), and two-sided *p* values. We chose to retain an explanatory variable in the final model if the p value was less than 0.10. For multinomial regression, we used the “nnet” package [20]. A two-sided p value < 0.05 was considered statistically significant. We used Eurostat’s European Standard Population (ESP2013) for age standardization [21].

## Results

The total number of deaths among Norwegian residents in 2005–2014 was 413,838, of which 24,963 (6.0%) had an external cause of death. The general characteristics of the data material are shown in Table 2.

**Table 2:** Characteristics of injury deaths in Norway, 2005–2014

	N (%)	Females (%)	Age (yrs) median (IQR)	Dying in health care institutions (%)	Main injury in hip/thigh region (%)
Road traffic accidents	2211 (8.9)	570 (25.8)	44 (25–64)	29.7	0.6
Accidental falls	4218 (16.9)	2082 (49.4)	85 (76–90)	82.6	31.6
< 70 yrs	737	160 (21.7)	57 (46–64)	51.0	4.1
≥ 70 yrs	3481	1922 (55.2)	87 (82–91)	89.2	37.4
Accidental poisonings	3329 (13.3)	882 (26.5)	42 (31–53)	14.2	0
Other accidents and events of undetermined intent	2930 (11.7)	942 (32.2)	61 (43–79)	36.7	1.3
Exposure to unspecified factor (X59)	6440 (25.8)	3970 (61.6)	88 (83–92)	93.1	79.2
Intentional self-harm (suicide)	5412 (21.7)	1574 (29.1)	45 (31–58)	10.4	0
Assault (homicide)	423 (1.7)	178 (42.1)	35 (21–51)	15.6	0.7
Total	**24,963 (100)**	**10,198 (40.9)**	**66 (42–86)**	**49.3**	**26.0**

Source: Norwegian Cause of Death Registry

### Predictors for use of the ICD-10 code X59

For the years 2005–2014, 6440 (1.6% of all deaths and 25.8% of the injury deaths) among Norwegian residents were coded with X59 (X59, X59.0, or X59.9) as the underlying cause of death. The results from the logistic regression models for each explanatory variable (unadjusted) are given in Table 3. All the investigated variables had a statistically significant association with X59, except calendar year of death. There was a predominance of women (OR 3.17, 95% CI 2.99–3.37) and persons of advanced age (85.6% of the persons with X59 were 80 years or older, compared to 22.5% in the group with known external cause of death). Seventy-nine percent had injuries in the hip or thigh region (OR 36.0, 95% CI 32.0–40.7). Fifty-five percent died in a nursing home (OR 40.8, 95% CI 35.9–40.7). Only 3.9% underwent an autopsy, and in 89.5% of the cases there was no information on the scene of injury. Based on these results, it seems like the typical X59 death occurred in an elderly woman with an injury (fracture) in the hip or thigh region, dying in a nursing home. In the multiple predictors model, also shown in Table 3, we found that the strongest predictor was the nature of injury, followed by lack of knowledge about the scene of injury.

**Table 3:** Predictors for X59-coded deaths

Explanatory variable	All external cause (%) N = 24,963	X59 (%) *N* = 6440	Not X59 (%) *N* = 18,523	Single predictor models				Multiple predictor model			
				OR (95% CI)		LR stat*	*P* value	OR (95% CI)		LR stat*	*P* value
Gender						1537	< 0.001			4.9	0.03
Male	14,765 (59.1)	2470 (38.4)	12,295 (66.4)	1 (ref.)				1 (ref.)			
Female	10,198 (40.9)	3970 (61.6)	6228 (33.6)	3.17	(2.99–3.37)			1.14	(1.01–1.27)		
Age						10,182	< 0.001			77.2	< 0.001
0–49 yrs	8219 (32.9)	64 (1.0)	8155 (44.0)	0.03	(0.02–0.03)			0.55	(0.37–0.82)		
50–59 yrs	2677 (10.7)	69 (1.1)	2608 (14.1)	1 (ref.)				1 (ref.)			
60–69 yrs	2100 (8.4)	143 (2.2)	1957 (10.6)	2.76	(2.07–3.72)			1.44	(0.99–2.11)		
70–79 yrs	2281 (9.1)	653 (10.1)	1628 (8.8)	15.2	(11.8–19.7)			1.89	(1.34–2.69)		
80–89 yrs	5858 (23.5)	3112 (48.3)	2746 (14.8)	42.8	(33.8–55.2)			2.00	(1.44–2.80)		
90+ yrs	3828 (15.3)	2399 (37.7)	1429 (7.7)	63.5	(49.9–82.0)			1.64	(1.17–2.32)		
Place of death						9331	< 0.001			182	< 0.001
At home	5889 (23.6)	312 (4.8)	5577 (30.1)	1 (ref.)				1 (ref.)			
Hospital	7222 (28.9)	2455 (38.1)	4767 (25.7)	9.21	(8.15–10.4)			0.63	(0.51–0.78)		
Nursing home	5088 (20.4)	3538 (54.9)	1550 (8.4)	40.8	(35.9–46.5)			1.22	(0.97–1.52)		
Other known	5020 (20.1)	44 (0.7)	4976 (26.9)	0.16	(0.11–0.22)			0.21	(0.14–0.31)		
Unknown	1744 (7.0)	91 (1.4)	1653 (8.9)	0.98	(0.77–1.25)			0.46	(0.32–0.66)		
Autopsy						6792	< 0.001			51.9	< 0.001
No autopsy	14,534 (58.2)	6189 (96.1)	8345 (45.1)	1 (ref.)				1 (ref.)			
Forensic	9695 (38.8)	104 (1.6)	9591 (51.8)	0.02	(0.01–0.02)			0.43	(0.33–0.57)		
Medical	734 (2.9)	147 (2.3)	587 (3.2)	0.39	(0.28–0.41)			0.53	(0.40–0.72)		
Nature of injury						14,759	< 0.001			3787	< 0.001
Head/neck	4244 (17.0)	392 (6.1)	3852 (20.8)	1 (ref.)				1 (ref.)			
Thorax	1055 (4.2)	134 (2.1)	921 (5.0)	1.43	(1.16–1.76)			1.65	(1.26–2.17)		
Abdomen/pelvis	665 (2.7)	341 (5.3)	324 (1.7)	10.3	(8.61–12.4)			4.05	(3.17–5.19)		
Hip/thigh	6495 (26.0)	5103 (79.2)	1392 (7.5)	36.0	(32.0–40.7)			12.1	(10.4–14.2)		
Other mechanical injury	2427 (9.7)	436 (6.8)	1991 (10.7)	2.15	(1.86–2.49)			1.72	(1.42–2.09)		
Poisoning	5010 (20.1)	3 (0.05)	5007 (27.0)	0.006	(0.001–0.02)			0.009	(0.002–0.02)		
Suffocation/drowning	4132 (16.6)	6 (0.1)	4126 (22.3)	0.01	(0.006–0.03)			0.02	(0.006–0.03)		
Other/sequelae	935 (3.7)	25 (0.4)	910 (4.9)	0.27	(0.18–0.40)			0.07	(0.04–0.10)		
Scene of injury						9224	< 0.001			3184	< 0.001
Unknown	10,081 (40.4)	5762 (89.5)	4319 (23.3)	1 (ref.)				1 (ref.)			
Known	14,882 (59.6)	678 (10.5)	14,204 (76.7)	0.04	(0.03–0.04)			0.05	(0.04–0.05)		
Calendar year of death						0.008	0.93			3.1	0.07
2005–2009	12,265 (49.1)	3161 (49.1)	9104 (49.1)	1 (ref.)				1 (ref.)			
2010–2014	12,698 (50.9)	3279 (50.9)	9419 (50.9)	1.00	(0.95–1.06)			0.91	(0.83–1.01)		

Logistic regression analysis, data from the Norwegian Cause of Death Registry, 2005–2014

*LR stat: Likelihood ratio statistic (−2 logL)

### Redistribution

**Fig. 2:**
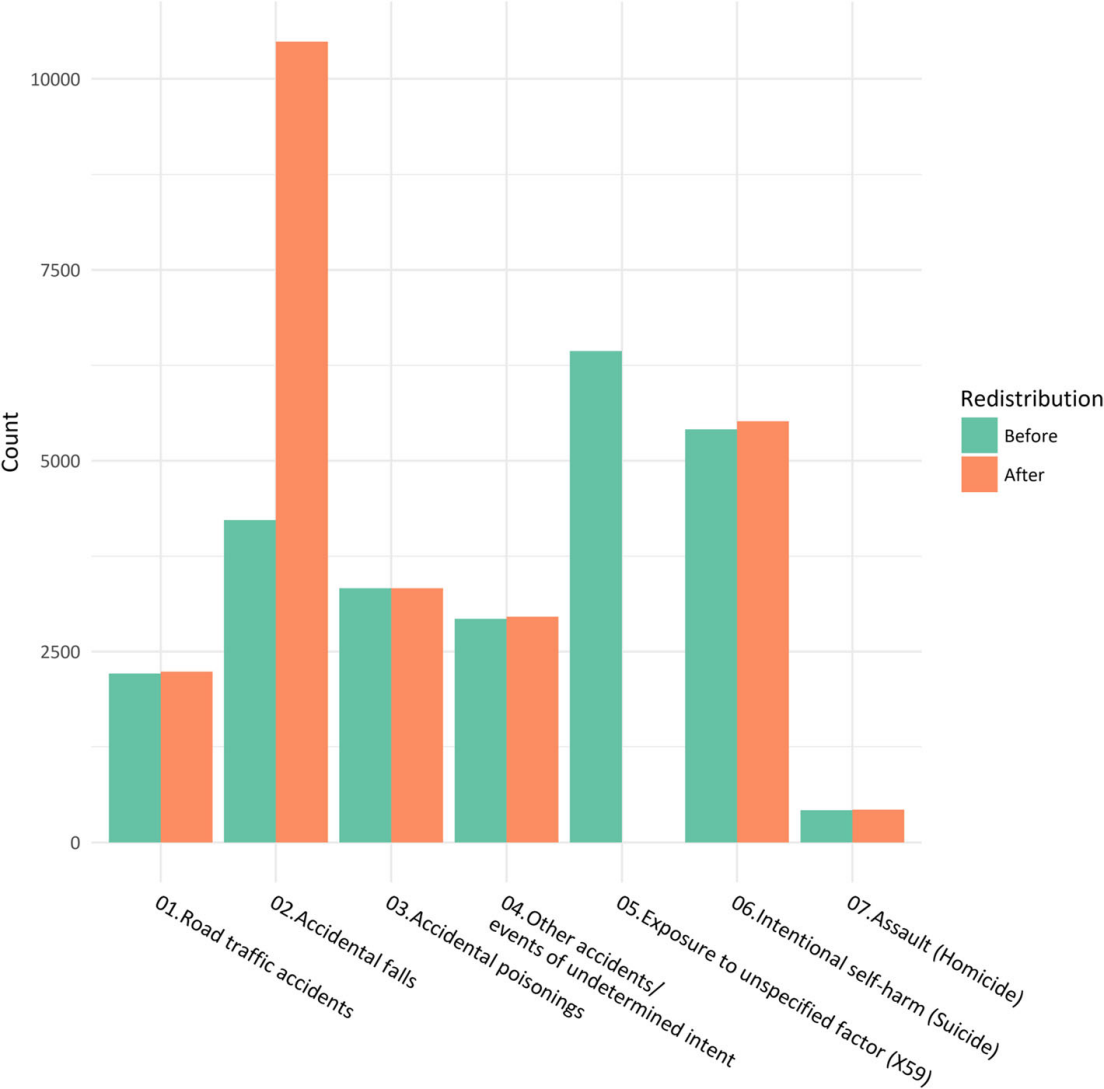
Results of redistribution of X59 cases in Norway, 2005–2014. Number of external causes of death in Norway, 2005–2014, before and after redistribution of X59 cases

We used multinomial logistic regression to redistribute X59 deaths to the most likely non-garbage code. We split the non-X59 cases into training and test sets and developed the regression model on the training set. The performance of the model was evaluated on the test set before we applied the model on the X59 cases. This procedure was repeated 1000 times. The median overall accuracy of prediction on the test set was 0.71, kappa 0.64. For the classification fall/not fall, the sensitivity was 0.85 and the specificity 0.96. The most important variables were the nature of injury, followed by the place of death and the age of the deceased (see Table 4). We found that almost all of the X59 cases (97.4%) were to be redistributed to accidental falls. This meant that for the 10-year study period, the number of deaths due to accidental falls increased by 148.7%, from 4218 to 10,490 deaths (Table 5 and Fig. 2). The mean age-standardized death rate from accidental falls for the years 2005–2014 increased from 10.3 per 100,000 to 25.9 per 100,000.

**Table 4:** Multinomial logistic regression model for redistribution of X59 deaths

Complete model	Accuracy (mean(SD))	Kappa (mean(SD))	LR stat (mean(SD))*	p value
	0.712 (0.01)	0.636 (0.01)	Ref.	
Reduced models				
*Without (one at a time)*				
Nature of injury	0.494 (0.01)	0.341 (0.01)	10,582 (147)	< 0.001
Place of death	0.673 (0.01)	0.586 (0.01)	1563 (61)	< 0.001
Age	0.690 (0.01)	0.607 (0.01)	1298 (56)	< 0.001
Autopsy	0.706 (0.01)	0.629 (0.01)	366 (26)	< 0.001
Scene of injury	0.704 (0.01)	0.627 (0.01)	309 (27)	< 0.001
Gender	0.712 (0.01)	0.636 (0.01)	199 (22)	< 0.001
Calendar year of death	0.711 (0.01)	0.635 (0.01)	33 (8)	< 0.001

Data from the Norwegian Cause of Death Registry, 2005–2014

Based on 1000 repetitions of random division into new training and test sets. The models were developed on the training sets and evaluated on the test sets

*The likelihood ratio statistic (−2 logL) is computed by comparing the full model to the model *without* the variable in question. The higher the LR statistics, the more the model is weakened by excluding the variable in question

**Table 5:** Results of redistribution of X59 deaths to specific external cause groups

	Before redistribution	Number redistributed (mean [SD])	% of X59	After redistribution	Change %	Sensitivity (mean [SD])	Specificity (mean [SD])
Total	18,523	6440	100.0	24,963			
Road traffic accidents	2211	24.2 (1.10)	0.4	2235.2	1.1	0.60 (0.01)	0.96 (< 0.01)
Accidental falls	4218	6271.9 (2.26)	97.4	10,489.9	148.7	0.85 (0.01)	0.96 (< 0.01)
Accidental poisonings	3329	3.0 (0.00)	0.0	3332.0	0.1	0.69 (0.01)	0.99 (< 0.01)
Other accidents and events of undetermined intent	2930	27.6 (0.50)	0.4	2957.6	0.9	0.73 (0.02)	0.91 (< 0.01)
Suicide	5412	107.8 (2.34)	1.7	5519.8	2.0	0.67 (0.01)	0.84 (< 0.01)
Homicide	423	5.6 (1.17)	0.1	428.6	1.3	0.41 (0.06)	0.98 (< 0.01)

Data from the Norwegian Cause of Death Registry 2005–2014

Based on 1000 repetitions of multinomial logistic regression. Overall accuracy 0.71 (0.01), kappa 0.64 (0.01) (mean [SD])

All cases except one (5102 of 5103) with hip and thigh injuries were redistributed to accidental falls. For the cases redistributed to accidental falls, the median age was 88 years and 63% were women. In comparison, for those being redistributed to road traffic accidents, the median age was 57 years and 21% were women, and for suicides the median age was 57 years and 15% were women. Further details of the redistribution results are given in Table 6.

**Table 6:** Detailed results of redistribution of X59 deaths to specific external cause groups

	N	Traffic accidents (mean [SD])	Accidental falls (mean [SD])	Accidental poisonings (mean [SD])	Other/undetermined (mean [SD])	Suicide (mean [SD])	Homicide (mean [SD])
Nature of injury							
01.Head/neck	392	14.2 (0.75)	319.6 (1.14)	0 (0)	0 (0.03)	56.1 (0.85)	2.1 (0.37)
02.Thorax	134	1.4 (0.48)	125.5 (0.73)	0 (0)	0 (0)	6.2 (0.93)	1.0 (0.04)
03.Abdomen/pelvis	341	1.1 (0.24)	334.6 (0.96)	0 (0)	0 (0)	3.3 (1.37)	2.0 (0.91)
04.Hip/thigh	5103	0 (0)	5101.9 (0.36)	0 (0)	0.6 (0.50)	0 (0)	0.5 (0.69)
05.Other mechanical injury	436	7.5 (0.64)	390.2 (1.20)	0 (0)	0 (0)	38.3 (1.02)	0 (0)
06.Poisoning	3	0 (0)	0 (0)	3.0 (0)	0 (0)	0 (0)	0 (0)
07.Suffocation/drowning	6	0 (0)	0 (0)	0 (0)	2.0 (0)	4 (0)	0 (0)
08.Other	25	0 (0)	0 (0)	0 (0)	25.0 (0)	0 (0)	0 (0)
Age group							
0–49 years	64	10.2 (0.4)	8.2 (0.84)	2.0 (0)	4.0 (0.03)	36.7 (1.15)	3.0 (0.85)
50–69 years	212	9.8 (0.76)	146.8 (1.39)	1.0 (0)	3.6 (0.50)	48.2 (1.26)	2.6 (0.86)
70+ years	6164	4.2 (0.68)	6116.9 (1.35)	0 (0)	20.0 (0)	23.0 (1.25)	0 (0)
**Total**	**6440**	**24.2 (1.13)**	**6271.8 (2.19)**	**3.0 (0)**	**27.6 (0.50)**	**107.9 (2.28)**	**5.6 (1.26)**

Data from the Norwegian Cause of Death Registry, 2005–2014

Based on 1000 repetitions of multinomial logistic regression

### Query to the certifying doctors

Of the 40,686 deaths among Norwegian residents in 2015, 3539 had an injury mentioned on the death certificate, either in part I or part II. For the X59 cases, we sent 559 query letters to the certifying doctors, either directly or via the chief municipal medical officer (Fig. 1). We identified 32 additional cases as previously described, making a total of 591 cases and 1.5% of all deaths among Norwegian residents this year. The median age among the cases was 88 years, with an interquartile range of 9, and 339 (57.3%) were women. Of the total, 433 (73.3%) had a hip or thigh injury, 539 (96.0%) died in a health care institution, and only 10 (1.7%) underwent an autopsy.

**Fig. 3:**
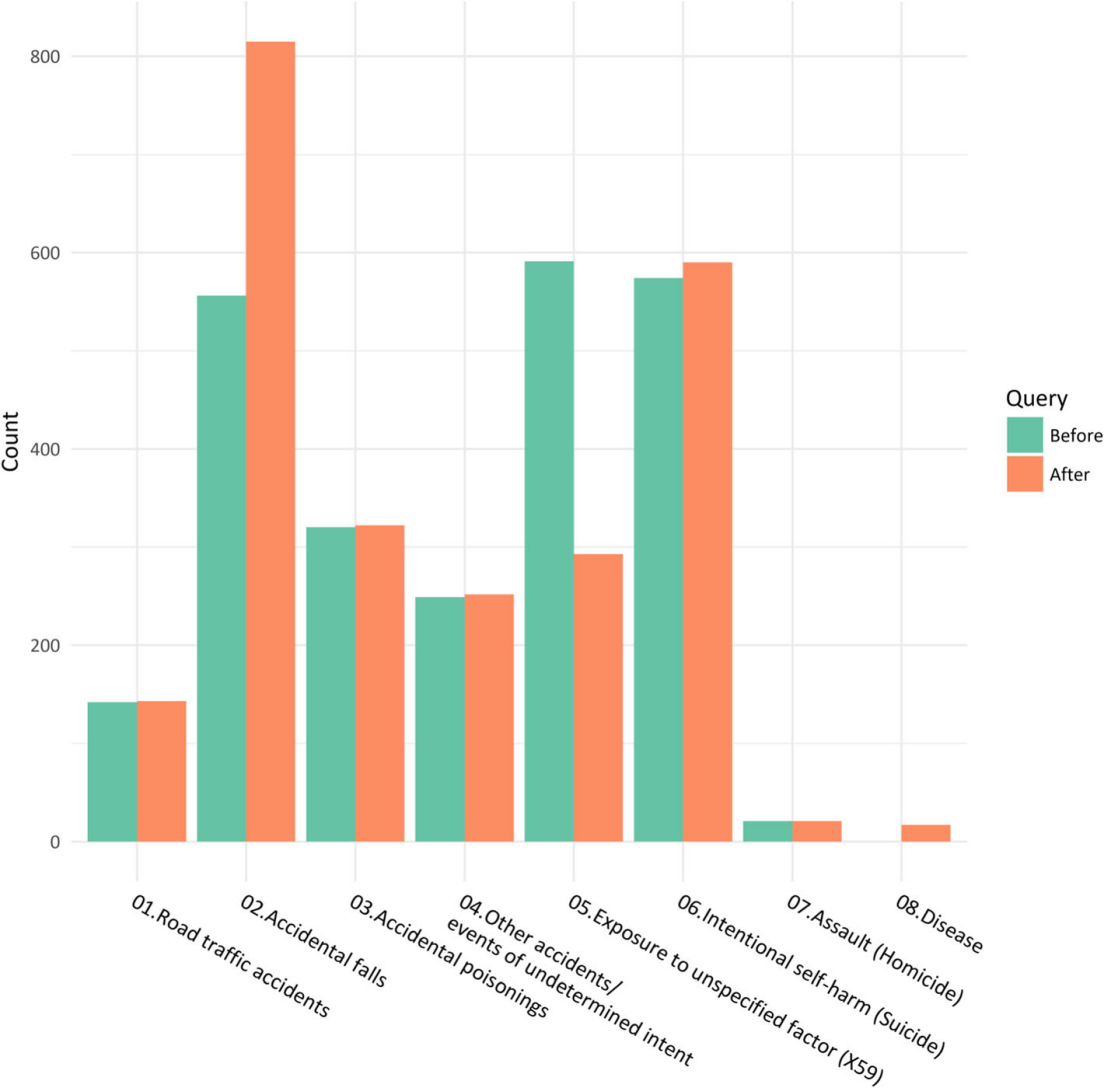
Results of query to the certifying doctors regarding X59 cases in Norway, 2015. Number of external causes of death in Norway 2015 before and after querying the X59 cases. The total value for cases with a non-injury cause of death is not shown in the graph, only the number allocated after the query project

The response rate was 67.8%. Eighty-eight (23.2% of the 379 replies) did not give any useful information, but 291 cases (76.8%) could be assigned a new and more specific underlying cause of death. The cause in the majority of these cases (257 of 291, 88.3%) was accidental falls. Altogether, we could reclassify 298/591 (50.4%) of the X59 cases. For details on the revised causes of death, see Table 7 and Fig. 3.

**Table 7:** Results from the X59 query at the Norwegian Cause of Death Registry for the 2015 data year

Revised cause of death	Reply received, *N* = 379	Reply not received, *N* = 180
Non-injury cause of death	15	2
Road traffic accidents	1	0
Accidental falls	257	2
Accidental poisonings	0	2
Other accidents and events of undetermined intent	3	0
Intentional self-harm (suicide)	15	1
Assault (homicide)	0	0
Not reclassified	88	173

In addition, there were 32 cases where no query was sent

In the group where the quality assurance process gave a new underlying cause of death (298 cases), taking into account the nature of the injury, we established that 258 out of 284 (90.8%) with a mechanical type of injury (S00-T14.9) died from an accidental fall. In the group with hip/thigh injuries (ICD-10 code S70-S79.9) 202 out of 214 (94.4%) were reclassified to falls.

### Use of X59 in the WHO mortality database

We analyzed data from the WHO Mortality Database for the years 2005–2014. Causes of death at the ICD-10 three- or four-character level were available for 125 countries and territories for at least one of the years. The fraction of all external causes of death coded with X59 varied from 0 to 42.1%, mean 6.4%. Use of X59 was most prevalent in Georgia (42.1%), Italy (31.4%), and Norway (26.0%). In eight locations, the X59 fraction was more than 20%, and in 76 locations below 5%. The fraction of all external causes of death coded with Y34 varied from 0 to 82.6%, highest in Azerbaijan (82.6%), Maldives (76.2%), and Bosnia and Herzegovina (54.4%). In Norway, the Y34 fraction was 0%. No countries had both a high X59 fraction and a high Y34 fraction.

## Discussion

We used data from the Norwegian Cause of Death Registry for the years 2005–2014 to investigate predictors for a death to be assigned the ICD-10 code X59 ("Exposure to unspecified factor") as the underlying cause of death. One-quarter of the deaths due to external causes lacked information on the circumstances leading to the fatal injury. Using data from the WHO Mortality Database, we showed that Norway is among the countries with the most prevalent use of X59 in external causes. For Norway, we developed a multinomial logistic regression model to reclassify the X59 deaths to a specific external cause group. Using this model, we estimated that 97% of the X59 deaths were accidental falls. We also sent query letters to the certifying doctors regarding the X59 cases in 2015. In 88% of the cases where we could assign a more specific external cause of death, this was an accidental fall.

Although our study is limited to the ICD-10 period in Norway, it is useful to compare the function and place in the classification system for X59 with similar codes in the previous ICD revision. In ICD-9, the code E887 (“Fracture, cause unspecified”), was used in cases where there was information that there had been a fracture, but without information on the circumstances around the injury. E887 was included in the “Falls” group and therefore often tabulated together with accidental falls. The ICD-10 code closest to E887 was initially X59 (“Exposure to unspecified factor”). Unlike E887, X59 included all kinds of injury and exposure, not only fractures. Also unlike E887, X59 is not included in the “Falls” group, but in the group “Accidental exposure to other and unspecified factors”. This could potentially lead to shifts in the total number of deaths classified as accidental falls. In an ICD-10 update in effect since 2006, X59 was subdivided into X59.0 (“Exposure to unspecified factor causing fracture”) and X59.9 (“Exposure to unspecified factor causing other and unspecified injury”). The code X59.0 would then include the same deaths as ICD-9 E887 (but not be included in the “Falls” group).

Norway has used ICD-10 for mortality coding since 1996. For the years 1996–2004, there was a national guideline stating that if a death certificate stated fracture of the femur (ICD-10 code S72) as the main injury, but without mention of the circumstances, the underlying cause of death should be coded as W19 (“Unspecified fall”). In 2005, this guideline was removed, and such cases would be assigned X59 as the underlying cause of death [22]. Similar rules for coding, tabulation, or presenting of statistics have been implemented in several countries, for instance Australia, to ensure continuity in the cause of death statistics [10].

### Redistribution of X59 deaths to a specific external cause

Several studies have directly or indirectly shown that a large proportion of unspecified injury deaths represent accidental falls in the elderly. Hu and Mamady found that in the US in 1999–2010 there was a clear negative correlation between the unspecified unintentional injury mortality in the elderly and the mortality from accidental falls [13]. During the study period, the proportion of unintentional injuries with unspecified circumstances (X59) decreased and the death rate from accidental falls increased. When they adjusted for the improved specificity of reporting of injury deaths, the increase of fall deaths was 61% instead of 77%. Gagné et al. also found a similar relationship in Quebec, Canada, for the years 2000–2009 [12]. The mortality rate for certified accidental falls in persons above 65 years increased and the rate for presumed falls (X59 as underlying cause of death plus mention of fracture on the death certificate) declined. The sum of the death rates due to certified and presumed falls was more or less stable in women and decreased slightly in men. In Australia, Harrison and Kreisfeld used the same definition of presumed fall and estimated that half of the deaths due to accidental falls were missing from the conventional cause of death statistics. The age distribution was similar to the conventional fall group, and 73% had hip fractures [10]. In Sweden, Johansson and Westerling found that the number of deaths due to accidental falls in Sweden in 1995 would increase by 57% if discharge information from hospitalizations within one year prior to death was added to the information on the death certificates [23].

Some of the X59-coded deaths in our study were redistributed to suicides, about 10 per year (1.7%), and two to three to road traffic accidents (0.4%). The rest, 0.5%, were distributed over the remaining groups. It is generally believed that official statistics miss some of the suicides, because of missing or incorrect information on the death certificates [24]. For example, the death may be classified as an accident instead of a suicide.

Bhalla and Harrison have expressed some concern that the Global Burden of Disease project redistributes too many deaths to road traffic accidents [25]. In our study, we have not found that a large proportion of X59 deaths represent road traffic accidents.

### Selection of target groups

In choosing the target groups for redistribution of X59, we have included all external causes, not only accidents. This means that some of the X59 cases might be redistributed to suicides or homicides. This is the same approach as in the Global Burden of Disease study, where the target groups for X59 are all injuries ([1]Appendix section 2.4). We did not include non-injury causes of death as target groups. When querying the certifying doctors, we realized that some of the injury deaths could have a disease as the underlying cause of death, and the injury was reclassified as a contributory cause of death. In the query, this occurred with 17 out of the 298 deaths (5.7%) where we could assign a new underlying cause of death. It is not always possible to decide whether a medical condition such as a myocardial infarction is a complication to the injury or a completely separate condition.

### Query to the certifying doctors

We found that for 88% of the X59 cases where we received additional information from the certifying doctors, the cause of death could be reclassified as accidental fall. This is slightly lower than the result from redistribution by regression (97%). We noted that a substantial number of the doctors did not regard a hip fracture as an accident and did not understand the purpose of our query about the circumstances of the injury. Many of the certifying doctors were either affiliated with nursing homes or were general practitioners on call, and had probably limited information about the event that had given rise to the injury.

There may be several explanations for missing information on the circumstances of an injury. Death due to a hip fracture often occurs days or weeks after the incident, perhaps at a nursing home or another institution. Thus, the doctor certifying the death may not have all the relevant information on the circumstances of the injury and the focus may be on the patient’s condition at the time close to death (the immediate cause of death), often a non-surgical complication, such as heart failure or pneumonia. Many elderly people have several diseases, and it can be difficult to decide which condition had the largest impact on the cause of death. Some doctors do not regard a low-level fall with a hip fracture as an accident or an external cause. In addition, many doctors are not fully familiar with the WHO instructions for cause of death certification.

### Strengths and limitations

A strength of this study is that it is population-based and includes all deaths with external causes among Norwegian residents for a recent 10-year period.

In contrast to the redistribution efforts by the Global Burden of Disease project [1], we had all the information on the death certificates available when we developed the redistribution model. Especially information on the nature of injury and the place of death contributed to the classification. This made it possible to perform redistribution on individual-level instead of group-level estimates. The World Health Organization also has a similar group-level approach in estimating causes of death but does not include X59 as an ill-defined code to be redistributed [26].

Even if the overall accuracy and kappa value for the redistribution model were 0.71 and 0.64, respectively, the discriminatory performance for the distinction “fall/not fall” had a sensitivity of 0.85 and specificity 0.96. The results were stable when we repeated the calculations 1000 times with new training and test sets.

Another strength of our study is that in addition to the analyses on available registry data, we performed a query of the certifying doctors. The results of this query strongly support the findings from the redistribution by multinomial logistic regression. A limitation of the query is that we received useful additional information in only 49.2% (291/591) of the X59 cases identified.

### Generalizability and implications

We believe that our approach could be used in other countries. Multinomial logistic regression is a well-known method for classification, and the procedure with splitting the data in a training and a test set, developing the model on the training set, and validating it on the test set is a recognized approach [18]. The exact importance of the different variables (and which variables should be included in the final model) and the performance of the model will vary among locations. The model must therefore be customized and evaluated in the specific setting where it is to be used. The results from the redistribution will also vary according to the local pattern of use of X59 (or other uninformative codes). One cannot directly claim from our observations in Norway that the majority of X59-coded deaths generally represent accidental falls. In other countries, a substantial part of X59-coded deaths might well be in another age segment and represent different causes of death than in Norway.

Our findings strongly suggest that the mortality from accidental falls is underestimated in official Norwegian statistics. Based on our estimates, the number of deaths due to falls in the study period should be nearly 150% higher than the official figures, and the actual death rate due to accidental falls among Norwegian residents should be about 25 deaths per 100,000 population, instead of the recorded 10.3/100,000. To reduce the number of X59 deaths and achieve more correct statistics, it is important to have efficient routines for querying the certifying doctors.

## Conclusions

One-quarter of the death certificates for Norwegians with an external cause of death lacked information on the circumstances leading to the injury. This is a serious flaw in the cause of death statistics. The majority of these cases were elderly women with hip injuries, dying in nursing homes. Both in redistribution with regression methods and in a query to the certifying doctors we found that almost all of these cases of X59-coded deaths represented accidental falls.
